# Trimolecular complex between major birch pollen allergen, Bet v 1, monoclonal allergen-specific human IgE and recombinant CD23

**DOI:** 10.1186/2045-7022-4-S2-P33

**Published:** 2014-03-17

**Authors:** Regina Selb, Julia Eckl-Dorna, Christian Lupinek, Birgit Linhart, Andrea Teufelberger, Walter Keller, Rudolf Valenta, Verena Niederberger

**Affiliations:** 1 Department of Otorhinolaryngology, Medical University of Vienna, 8H1.02, Waehringer Guertel 18-20, Vienna, 1090, Austria; 2 Medical University of Vienna, Department of Otorhinolaryngology, Vienna, Austria; 3 Medical University of Vienna, Department of Pathophysiology and Allergy Research, Vienna, Austria; 4 Karl Franzens University Graz, Institute of Molecular Biosciences, Graz, Austria

## 

CD23 the low affinity receptor for IgE plays an important role in allergic disease because it facilitates allergen presentation to T cells. CD23 is a 45 kDa trans-membrane protein consisting of an alpha-helical stalk and a head domain, which is mainly expressed on B cells. In order to analyze the complex formation between allergen, allergen-specific IgE and CD23 on the molecular level, we expressed four different forms of CD23 in baculovirus-infected SF9 insect cells. Construct A consists of the extracellular part of the molecule, the second construct (B) differs from construct A by a single amino acid exchange in the stalk region to abolish the N-linked glycosylation of CD23. Constructs C and D were smaller molecules consisting mainly of the head domain of the protein. Furthermore we expressed human monoclonal IgE specific for the major birch pollen allergen Bet v 1 in a hybridoma cell line and the Bet v 1 allergen in Escherichia coli.

Using circular dichroism analysis we found that all molecules were expressed as folded proteins and gel filtration revealed that the CD23 constructs were monomeric. In ELISA we demonstrated that each of the four CD23 constructs binds monomeric IgE and allergen-IgE complexes in a similar way. Concerning the different CD23 constructs, we observed weaker binding signals when measuring the smaller CD23 molecules. Interestingly, construct B, which was devoid of the N-linked glycosylation site, displayed enhanced IgE binding compared to the glycosylated protein.

In conclusion, our results reveal a comparable interaction between isolated IgE as well as IgE-allergen immune complexes with CD23 and provide defined molecules for the detailed structural characterization of the allergen-IgE-CD23 interaction.

Supported by grants 4605, 4613 and in part by 4604 and P23350-B11 of the Austrian Science Fund (FWF).

